# Development of SDP0505: a first-in-class HER3 × c-Met bispecific ADC, demonstrates potent antitumor activity in EGFR TKI-resistant NSCLC, CRC, and beyond

**DOI:** 10.1093/abt/tbag015

**Published:** 2026-04-21

**Authors:** Li-Min Wang, Changyong Yang, Simeng Chen, Xing Sun, Yunan Tian, Jieqiong Zhang, Xiaomeng Gao, Dan Li, Wei Zhang, Yali Hu, Jimin Yuan, Qumiao Xu, Xiyang Liu, Long Zhang, Qianqian Yu, Cheng Liao

**Affiliations:** Jiangsu Hengrui Pharmaceuticals Co., Ltd, Jiangsu 222047, China; Jiangsu Hengrui Pharmaceuticals Co., Ltd, Jiangsu 222047, China; Jiangsu Hengrui Pharmaceuticals Co., Ltd, Jiangsu 222047, China; Jiangsu Hengrui Pharmaceuticals Co., Ltd, Jiangsu 222047, China; Jiangsu Hengrui Pharmaceuticals Co., Ltd, Jiangsu 222047, China; Jiangsu Hengrui Pharmaceuticals Co., Ltd, Jiangsu 222047, China; Jiangsu Hengrui Pharmaceuticals Co., Ltd, Jiangsu 222047, China; Jiangsu Hengrui Pharmaceuticals Co., Ltd, Jiangsu 222047, China; Jiangsu Hengrui Pharmaceuticals Co., Ltd, Jiangsu 222047, China; Jiangsu Hengrui Pharmaceuticals Co., Ltd, Jiangsu 222047, China; Jiangsu Hengrui Pharmaceuticals Co., Ltd, Jiangsu 222047, China; Jiangsu Hengrui Pharmaceuticals Co., Ltd, Jiangsu 222047, China; Jiangsu Hengrui Pharmaceuticals Co., Ltd, Jiangsu 222047, China; Jiangsu Hengrui Pharmaceuticals Co., Ltd, Jiangsu 222047, China; Department of Tumor Biobank, Shanxi Province Cancer Hospital/ Shanxi Hospital Affiliated to Cancer Hospital, Chinese Academy of Medical Sciences/Cancer Hospital Affiliated to Shanxi Medical University, Taiyuan, Shanxi 030013, China; Jiangsu Hengrui Pharmaceuticals Co., Ltd, Jiangsu 222047, China

**Keywords:** IHC staining, EGFR TKI resistant, HER3, c-Met, bispecific ADC, LUAD

## Abstract

**Background:**

Despite the success of third-generation epidermal growth factor receptor (EGFR) tyrosine kinase inhibitors (TKIs) in EGFR-mutant non-small cell lung cancer (NSCLC), acquired resistance remains an unmet need. Although human epidermal growth factor receptor 3 (HER3) and c-Met upregulation are key mechanisms of this resistance, the rationale for simultaneously targeting both to overcome this challenge remains unexplored.

**Methods:**

HER3 and c-Met expression were evaluated by immunohistochemistry in 270 clinical samples, including EGFR TKI-resistant NSCLC, lung adenocarcinoma (LUAD), gastric cancer, and colorectal cancer (CRC). The binding affinity of SDP0505 was measured by surface plasmon resonance and enzyme-linked immunosorbent assay, internalization was monitored using Incucyte, and *in vitro* cytotoxicity was assessed via CellTiter-Glo assay. The *in vivo* anti-tumor activities were investigated in cell-derived and patient-derived xenograft (PDX) models. Pharmacokinetics and repeated-dose toxicity were studied in cynomolgus monkeys.

**Results:**

Immunohistochemical analyses revealed spatial colocalization of HER3 and c-Met in EGFR TKI-resistant NSCLC specimens. Furthermore, high co-expression was observed in 58% of EGFR-mutant LUAD and 95% of CRC cases. Consequently, SDP0505 was developed with optimal antigen-binding affinity and antibody format through comprehensive screening. In HER3/c-Met dual-positive cell lines, SDP0505 demonstrated enhanced cell binding and internalization over the in-house synthesized U3-1402 analog. Superior *in vivo* antitumor efficacy was also observed in multiple NSCLC and CRC models, including EGFR TKI-resistant PDX models. Moreover, SDP0505 exhibited favorable pharmacokinetic and safety profiles in cynomolgus monkeys.

**Conclusions:**

SDP0505 represents a novel HER3 × c-Met ADC with superior internalization and anti-tumor activity in preclinical models, especially in EGFR TKI-resistant PDX models. These results supported the initiation of a Phase I clinical trial in China.

## Teaser

Statement of Significance We developed SDP0505, a first-in-class HER3/c-Met bispecific ADC, based on prevalent co-expression of both targets in numerous cancers, especially in EGFR TKI-resistant NSCLC. SDP0505 exhibited enhanced internalization and superior antitumor activity over the HER3-targeting ADC benchmark in EGFR TKI-resistant PDX models, supporting its clinical translation.

## Introduction

Lung cancer persists as the leading cause of cancer-related mortality worldwide, with non-small cell lung cancer (NSCLC) accounting for ~85% of all cases [1]. As the primary oncogenic driver, epidermal growth factor receptor (EGFR) mutations are found in 51.0% of global and 53.0% of Asian non-squamous NSCLC patients [2]. Although third-generation EGFR tyrosine kinase inhibitors (TKIs) like osimertinib have revolutionized the treatment of advanced EGFR-mutant NSCLC [3], acquired resistance inevitably develops through complex and poorly characterized mechanisms compared to those of earlier-generation TKIs [4].

Within the spectrum of EGFR-independent resistance mechanisms, compensatory bypass signaling pathways have been identified as pivotal contributors, with growing evidence implicating dysregulation of mesenchymal-epithelial transition factor (*MET*) and human epidermal growth factor receptor 3 (*HER3*) as key molecular drivers. *MET* amplification, observed in 15%–19% of EGFR TKI -resistant tumors, is the predominant EGFR-independent resistance mechanism [5]. Concurrently, acquired resistance to EGFR TKI in EGFR-mutant NSCLC correlated with a 1.5-fold upregulation of cell-surface HER3 expression relative to treatment-naïve states [6]. Thus, targeting HER3 or c-Met represents a mechanistically rational strategy to overcome EGFR TKI resistance. This strategy has gained clinical validation through two distinct modalities: HER3-directed antibody-drug conjugate (ADC) and EGFR-MET bispecific antibody. U3-1402 (patritumab deruxtecan), a HER3-targeted ADC, demonstrated promising antitumor activity in heavily pretreated EGFR-mutated, EGFR TKI-resistant NSCLC patients (median of three prior therapies), achieving an objective response rate (ORR) of 29.8% [7]. Notably, the EGFR-MET bispecific antibody amivantamab (RYBREVANT®) demonstrated enhanced efficacy when combined with carboplatin/pemetrexed in EGFR TKI-resistant NSCLC. In MARIPOSA-2 study, the combination regimen significantly prolonged progression-free survival (median PFS 6.3 months, 95% CI: 5.6–8.4) compared to chemotherapy alone (median PFS 4.2 months, 95% CI: 4.0–4.4), with a hazard ratio (HR) of 0.48 (*P* < .001) representing a 52% reduction in progression or death risk. Based on this evidence, both the FDA and China's National Medical Products Administration (NMPA) have approved this regimen for EGFR-mutant (ex19del/L858R) NSCLC progressing after EGFR TKI therapy [8].

Over the past decade, ADCs have evolved into a cornerstone of precision oncology, demonstrating transformative potential in the management of both solid and hematologic malignancies. Despite remarkable progress in ADC development, significant challenges persist, including constrained clinical efficacy, limited improvements in toxicity profiles relative to the payload [9], and the emergence of resistance mechanisms such as antigen downregulation or activation of compensatory pathways [10]. Bispecific ADCs (BsADCs) address these limitations by simultaneously engaging co-expressed antigens, enhancing tumor specificity, overcoming resistance through redundant targeting, improving internalization efficiency, and expanding the therapeutic window [11, 12]. Utilizing HER3 as a novel target to overcome EGFR resistance, Baili Biopharma developed BL-B01D1, a first-in-class EGFR×HER3 BsADC that has been designated as a groundbreaking therapeutic candidate by the NMPA. In clinical studies, BL-B01D1 demonstrated an ORR of 52.5% in EGFR-mutated NSCLC. Notably, the drug has advanced to global Phase II clinical trials for NSCLC, with China's BLA submission targeted for 2026 [13].

Although HER3- and c-Met-targeted agents have been individually employed in the treatment of EGFR TKI-resistant patients [7, 8], the therapeutic potential of dual-targeting both HER3 and c-Met in this population remains unexplored. Our immunofluorescence (IF) analysis of EGFR TKI-resistant clinical specimens revealed, for the first time, spatial colocalization of HER3 and c-Met proteins within the same tumor regions in the single-cell level. This novel observation provides first IF rationale for developing combination therapies that simultaneously targeting both targets to overcome TKI resistance. Here, we describe the discovery and development of SDP0505, a novel BsADC simultaneously targeting c-Met and HER3. SDP0505 comprises an optimized bispecific antibody conjugated to SHR9265, a topoisomerase I (Topo I) inhibitor, via a cleavable linker, with a targeted drug-to-antibody ratio (DAR) of 6.0. Preclinical studies revealed enhanced internalization and potent antitumor activity compared to U3-1402 (in-house synthesized analog) across NSCLC and CRC models, especially in EGFR TKI-resistant patient-derived xenograft (PDX) models. In addition, SDP0505 exhibited favorable pharmacokinetic characteristics and acceptable safety profiles in cynomolgus monkeys. Based on these promising results, SDP0505 is currently under investigation in a Phase I trial in China.

## Materials and methods

### Cell lines, materials, and test substances

HCC827, NUGC-4, NCI-H441, PC-9, NCI-H1703, SW620, Raji, and NK92-CD16A-176 V were provided by Shanghai Hengrui Pharmaceutical Co., Ltd. NCI-H522 and NCI-H358 were purchased from ATCC.

SDP0505 was provided by Suzhou Suncadia Biopharmaceuticals Co., Ltd. hIgG1 was supplied by Shanghai Hengrui Pharmaceutical Co., Ltd. U3-1402 analog was produced by ChemPartner based on the publicly available sequence and chemical structure. SDP01867 (anti-HER3) and SDP01869 (anti-c-Met) were synthesized by GenScript according to the amino acid sequences we provided.

### Immunohistochemistry (IHC)

Formalin-fixed and paraffin-embedded Patient tumor samples were obtained from Shanxi Province Cancer Hospital. All tumor samples were obtained in compliance with relevant laws and institutional guidelines, with approval from the ethics committees (22 February 2024, Approval No. KY2023042). The slides of formalin-fixed and paraffin-embedded tumors derived from patient-derived xenografts (PDXs) were obtained from LIDE Biotech. Adjacent tissue sections from the same tumor sample were used for HER3 and c-Met IHC staining, respectively, as described below. Firstly, the antigen retrieval of tissue sections was done by incubating the samples in Tris-EDTA (pH 9.0) for 20 min at 100°C. Endogenous peroxidase activity was quenched by incubation of the samples in pre-primary peroxidase inhibitor (BOND™ Polymer Refine Detection; Leica Biosystems, Cat. # DS9800) for 5 min at ambient temperature (20–25°C). Paired sample slides were incubated with the HER3 primary antibody (CST, Cat. #12708) and MET primary antibody (CST, Cat. #8198), diluted to 1.2 μg/mL (1:50) and 0.08 μg/mL (1:625) respectively in BOND Primary Antibody Diluent (Leica Biosystems, Cat. # AR9352) at room temperature. The sample slides were then incubated with the secondary polymer anti-rabbit poly–horseradish peroxidase-IgG, followed by incubation with the 3,3′-diaminobenzidine tetrahydrochloride hydrate (DAB) chromogen (BOND™ Polymer Refine Detection; Leica Biosystems, Cat. # DS9800) for 10 min at room temperature. After washing off the excess DAB, sample nuclei were counterstained with hematoxylin (BOND™ Polymer Refine Detection; Leica Biosystems, Cat. # DS9800) for 5 min at ambient temperature. Images were scanned with UNIC PRECICE 500 scanner.

### Scoring of HER3 and c-met IHC

HER3/c-Met is mainly localized to the plasma membrane, which may present as membranous and cytoplasmic expression. All viable tumor cells on the entire slide are included in the scoring assessment. Positivity for HER3/c-Met is defined as tumor cells showing partial or complete membranous staining. The percentage of tumor cells with membranous staining at four different intensities is estimated:

0 = No staining.

1+ = Weak staining.

2+ = Moderate staining.

3+ = Strong staining.

The sum of all four percentages should be equal to 100%.

Percentage of tumor cells (0%–100%) at each intensity level (0, 1+, 2+, 3+) must be documented for each sample. The H-score is calculated from the total of each individual intensity of staining multiplied by the percentage of cells and all values are added to give the final score ≤ 300.

H-score = [(% 0+) ^*^0] + [(%1+) ^*^ 1] + [(%2+) ^*^ 2] + [(%3+) ^*^ 3].

### Immunofluorescence (IF) staining of tumor tissues

Standard procedures were used for IF staining. Primary antibodies for IF were HER3 primary antibody (CST, Cat. #12708) and MET primary antibody (CST, Cat. #8198). Secondary antibodies were HRP anti-rabbit (Reagent included in BOND Polymer Refine Detection Kit, Cat. #DS9800, Leica Biosystems). The AKOYA TSA Fluorescein System was used to detect HER3 and Met (#FP1487001KT for human HER3, and #FP1488001KT for human Met). DAPI (AKOYA, Cat. #FP1490) was used as counterstain. Sections were mounted with Prolong Diamond antifade mountant (Invitrogen, Cat. #P36961). Fluorescence imaging of tissue was done with the Multiphoton Confocal Microscope Zeiss LSM 710.

### The surface plasmon resonance (SPR) assay to measure the binding affinity of SDP0505 to HER3 and c-met, human FcRs, FcRn, and C1q proteins

For the binding affinity of SDP0505 to human, cynomolgus, and mouse c-Met proteins, as well as human, cynomolgus, mouse, and rat HER3 proteins, SDP0505 was diluted in HBS-EP+ buffer and captured on a Protein A sensor chip in Flow cell 2 (Fc2) at 10 μL/min for 30 s. For the binding affinity to rat c-Met (hFc) proteins, biotinylated SDP0505 was immobilized on a CAP chip at 20 μL/min for 60 s. Serial dilutions of each analyte (human, cynomolgus, and mouse c-Met proteins, as well as human, cynomolgus, mouse, and rat HER3 proteins) were injected over Flow cell 1 (Fc1) and Fc2 at 30 μL/min. Chip regeneration was performed using either Glycine 1.5 (30 s, 30 μL/min) or Biotin CAPture Regeneration Solution (50 s, 20 μL/min).

For the binding affinity of SDP0505 to human FcγRs and FcRn, human FcγRI, FcγRIIA (H167 and R167), FcγRIIB, FcγRIIIA (F176 and V176), FcγRIIIB, and FcRn proteins were captured on a CM5 chip pre-immobilized with an anti-histidine antibody in Fc2. SDP0505 was injected as analyte in serial dilutions across Fc1 and Fc2 at 30 μL/min, followed by regeneration with Glycine 1.5 (30 s, 30 μL/min).

Human C1q protein binding was evaluated by injecting human C1q proteins over a CM4 chip pre-immobilized with SDP0505 or human IgG1 at 30 μL/min, with association and dissociation phases each lasting 120 s. Regeneration was carried out using 3 M MgCl₂ (30 s, 30 μL/min).

All SPR data were acquired on a Biacore 8K Control Software and processed using the Biacore 8K Evaluation Software. Affinity constants (KD) were determined by fitting to a 1:1 Langmuir binding model or a steady-state affinity model.

### The enzyme-linked immunosorbent assay (ELISA) to assess the dual target binding ability of SDP0505 to HER3 and c-Met

To evaluate the concurrent binding ability of SDP0505 to HER3 and c-Met, a sequential ELISA was performed as follows: c-Met protein was diluted to 1 μg/mL in PBS, and added to a 96-well flat-bottom plate. The plate was incubated overnight at 4°C. After removing the coating solution, wells were washed with PBST (PBS + 0.05% Tween-20) for three times. Blocking was performed with 3% BSA /PBS for 1 h at 37°C, after which wells were washed twice with PBST. SDP0505, SDP0505 BsAb (the naked antibody), and hIgG1 (negative control) were serially diluted (3-fold gradient, starting at 100 nM) in 1% BSA/PBS. Diluted antibodies were added and incubated for 2 h at room temperature (RT). After washing with PBST for three times, biotinylated-HER3 protein (1 μg/mL in 1% BSA/PBS) was added and incubated for 1 h at RT. Wells were washed three times with PBST, followed by addition of streptavidin-HRP (1:5000 in 1% BSA/PBS) and incubation for 1 h at RT. After a final wash step, TMB substrate was added. Color reaction was monitored for 5–10 min at RT. Reactions were terminated with stop solution, and absorbance was measured at 450 nm using a microplate reader. Data were analyzed using GraphPad Prism.

### Fluorescence-activated cell sorting (FACS)

Adherent cells were washed once with PBS and detached using TrypLE Express enzyme. The cell suspension was centrifuged at 400 g for 5 min, and the supernatant was discarded. Cells were resuspended in fluorescence-activated cell sorting (FACS) buffer (2% FBS/PBS) and washed once. After counting, the cells were adjusted to a density of 2 × 10^6^ cells/mL. A 50 μL aliquot of this cell suspension was seeded into a 96-well round-bottom plate yielding 1 × 10^5^ cells/well. The antibodies SDP01867 (anti-HER3), SDP01869 (anti-c-Met) and hIgG1 were diluted in FACS buffer to a working concentration of 200 nM. The antibodies were added to each corresponding well at a final concentration of 100 nM. The plate was then gently mixed and incubated at 4°C for 1 h. Control groups included wells with only secondary antibody and wells without any antibody.

After primary antibody incubation, cells were washed by centrifugation at 400 g for 5 min. The supernatant was discarded, and cells were washed twice with FACS buffer. Cells were then resuspended in 50 μL FACS buffer containing PE-conjugated Goat anti-Human IgG Fc secondary antibody (1:50 dilution). The plate was incubated in the dark at 4°C for 30 min. After incubation, cells were washed twice with FACS buffer, followed by centrifugation at 400 g for 5 min each. Finally, cells were resuspended in FACS buffer for analysis.

For fluorescence quantification, BD Quantibrite™ Beads were reconstituted in 0.5 mL FACS buffer, vortexed, and 250 μL was transferred to blank wells on the sample plate. Fluorescence signals were acquired using a flow cytometer. Data were analyzed using FlowJo software to determine the geometric mean fluorescence intensity (gMFI). PE molecules per cell were quantified based on the BD Quantibrite™ Beads standard curve.

### Cell binding assay

Adherent cells were washed once with PBS and detached using TrypLE Express enzyme. The cell suspension was centrifuged at 400 g for 5 min, and the supernatant was discarded. Cells were resuspended in FACS buffer (2% FBS/PBS) and washed once. Cells were counted and adjusted to a density of 2 × 10^6^ cells/mL. A 50 μL aliquot was seeded per well in a 96-well round-bottom plate, yielding 1 × 10^5^ cells/well.

SDP0505, SDP0505 BsAb, U3-1402 analog, and hIgG1 were serially diluted in FACS buffer with 3-fold gradient, starting from 400 nM. For NCI-H1703 cells, 50 μL of diluted antibody was added per well, resulting in a maximum final concentration of 200 nM. For NUGC-4 and MDA-MB-453 cells, 100 μL was added per well, achieving a maximum final concentration of 266.67 nM. The plate was mixed gently and incubated at 4°C for 1 h. Control groups included wells with only secondary antibody and wells without any antibody.

After incubation, cells were washed by centrifugation at 400 g for 5 min. The supernatant was discarded, and cells were washed twice with FACS buffer. Cells were then resuspended in 50 μL FACS buffer containing Alexa Fluor® 647-conjugated Mouse Anti-Human IgG secondary antibody (1:50 dilution). The control group without any antibody was not added secondary antibody. The plate was incubated in the dark at 4°C for 30 min, washed twice and resuspended in FACS buffer, then subjected to a flow cytometer for fluorescence signals dectection. Data were analyzed using FlowJo software to determine the gMFI. Dose–response curves were plotted, and EC_50_ values were calculated using GraphPad Prism.

### 2D cytotoxicity assay for early antibody screening

A HER3 and c-Met positive cell line, PC-9 was used in this assay. Cells were seeded in 96-well plates at 600 cells per well. The following day, serially diluted ADCs were added in equal volumes. After 6 days of incubation, cell viability was assessed using the CellTiter-Glo Luminescent Cell Viability Assay (Promega, Cat# G7570) according to the manufacturer’s protocol. Viability was calculated as a percentage relative to untreated control cells.

### 3D spheroid cytotoxicity assay

Adherent cells were washed once with PBS and detached using 1 mL of trypsin. The cell suspension was centrifuged at 400 g for 5 min, and the supernatant was discarded. Cells were resuspended in culture medium and counted, then seeded into ultra-low attachment 96-well plates. The edge wells were filled with 200 μL PBS to minimize evaporation. Plates were incubated overnight to allow spheroid formation. On the second day, serial dilutions of SDP0505, SDP0505 BsAb, U3-1402 analog and DXh were prepared and added to each well with three replicates per concentration. Plates were incubated for 9–11 days, with a partial medium change performed at the mid-culture point (Day 5 or 6). At endpoint, 100 μL supernatant was removed from each well. Plates were equilibrated to room temperature, and 100 μL CellTiter-Glo® 3D reagent (Promega, Cat# G9681) was added per well. After 30 min of orbital shaking, lysates were transferred to opaque plates, and luminescence was measured using a multimode plate reader.

Cell viability was calculated as: Viability (%) = (Drug-treated signal / Control signal) × 100.

Dose–response curves and IC_50_ values were generated using GraphPad Prism.

### Internalization assay for early antibody screening with αHFc-CL-MMAE

The αHFc-CL-MMAE (Moradec, Cat# AH-102AE-50) and the test antibodies were mixed at equimolar concentrations in a 1:1 volume ratio and incubated at 37°C for 30 min. The mixture was then serially diluted in cell culture medium using a 3-fold gradient and added to cells plated the previous day at a density of 600 cells per well. Following a 6-day incubation at 37°C in a 5% CO₂ incubator, cell viability was assessed by CellTiter-Glo kit (Promega, Cat# G7570). Chemiluminescence was measured using a PerkinElmer ENVISION plate reader, and the data were analyzed to determine the EC_50_ and E_max_ values, with both expressed relative to the control group without antibody.

### Internalization assay

NUGC-4 and NCI-H1703 cell lines were trypsinized, counted, and resuspended in complete medium. Cell suspensions were seeded into 96-well plates at 6000 cells/well in 50 μL medium and incubated overnight at 37°C in a 5% CO_2_ incubator. Antibody staining solutions were prepared in complete medium at 2× final concentration by mixing SDP0505, SDP0505 BsAb, U3-1402 analog and hIgG1. Incucyte® Human Fabfluor-pH Red Antibody Labeling Dye (Catlog No. 4722, Sartorius) were added into each antibody staining solutions and incubated at 37°C for 15 min protected from light. After incubation, 50 μL of the 2× antibody-dye solution was added to each well of the cell plate (final volume was 100 μL/well), to get the desired final concentrations. The plates were immediately transferred to an Incucyte® live-cell analysis system maintained at 37°C with 5% CO_2_. Fluorescence images were acquired every 15 min for continuous 23 h. Fluorescence intensity data were analyzed and plotted using GraphPad Prism.

### Bystander killing assay

HER3/c-Met-high expressing NCI-H441 was identified as SDP0505-sensitive cell line in previous 3D spheroid cell viability assay. Conversely, HER3/c-Met-negative NCI-H522 is identified as SDP0505-insensitive cell line. These two cell lines were trypsinized to single-cell suspensions and centrifuged at 400 g for 5 min. After one wash with PBS, cells were resuspended in PBS at 1 × 10^6^ cells/mL and differentially labeled NCI-H441 with CellTrace™ Violet (CTV) and NCI-H522 with CellTrace™ CFSE (CFSE). Cells were incubated with dyes in PBS at 37°C for 15 min. Staining was quenched with complete medium, followed by centrifugation at 400 g for 5 min and two washes with PBS to remove the extra dye.

The CTV-labeled NCI-H441 and CFSE-labeled NCI-H522 were mixed and seeded in 24-well plates at the density of 5 × 10^4^ and 1 × 10^4^ cells per well respectively. The NCI-H522 was also seeded alone at the density of 1 × 10^4^ as a negative control. Cells were incubated overnight at 37°C in 5% CO₂. On the next day, antibody solutions were prepared in complete medium and added into cells at the final concentration of 0.5 and 5 nM for SDP0505 and 5 nM for U3–1402 analog. After incubation for 5 days with medium replaced on Day3, cells were trypsinized and analyzed by flow cytometry. Data was plotted by GraphPad Prism.

### Antibody-dependent cellular cytotoxicity (ADCC) assay

CFSE-labeled NCI-H441 target cells were prepared at the density of 1.5 × 10^5^ cell/mL in 96-well ultra-low attachment plates. These cells were co-cultured with NK92-CD16A-176 V effector cells at an effector-to-target ratio of 5:1. Test antibodies (SDP0505, SDP0505 BsAb, hIgG1 isotype control) were serially diluted five-fold from a maximum final concentration of 50 nM. Parallel assays were performed using Raji cells treated with the same dilutions of rituximab and IgG1 as positive controls. Following incubation at 37°C for 4 h, cells were stained with propidium iodide (PI) and analyzed by flow cytometry to determine specific cytotoxicity, calculated as the percentage of CFSE^+^PI^+^ cells within the total CFSE^+^ population. Dose–response curves were generated using GraphPad Prism.

### Complement dependent cytotoxicity (CDC) assay

NCI-H441 cells were seeded into a transparent-bottom, white-edge 96-well plate at 4 x 10^5^ cell per well. Cells were treated with five-fold serial dilutions of SDP0505, SDP0505 BsAb, hIgG1 isotype control, with a maximum final concentration of 1 μM. Parallel positive control assays were performed using Raji cells treated with rituximab and IgG1. After treatment for 4 h, cell viability was quantified using the Cell Counting-Lite 2.0 Luminescent Assay according to manufacturer’s protocol. Luminescence was measured using a multi-function plate reader after 10 min of orbital shaking at room temperature (Perkin Elmer).

### In vitro stability assay in plasma from different species

The stability of SDP0505 was evaluated by quantifying the release of its conjugated payload (DXh) in plasma from CD-1 mouse, SD rat, cynomolgus monkey, and human with 5% BSA/PBS as a control. Samples were incubated at 37°C for up to 21 days. Free DXh in plasma and 5%BSA-PBS was measured at designated time points using LC–MS/MS. The percentage of toxin release was determined by comparing the amount of measured free DXh to the theoretical total amount of DXh payload conjugated to SDP0505.

### Cell-line derived and patient-derived xenograft studies

The study utilized 6–8 weeks old female Balb/c Nude mice in CDX models and NU/NU female mice in PDX models. Up to five mice were housed per sterilized cages under specific pathogen-free conditions, with temperatures maintained between 20°C and 26°C, relative humidity set between 30% and 70%, and a lighting schedule of 12 h of light followed by 12 h of darkness.

Throughout the experiments, tumor volumes and the body weights were measured twice a week. Tumor volume was calculated using the formula: tumor volume = (length × width^2^)/2. Relative Tumor Volume (RTV) was determined using: RTV = (V_t_-V_0_) / V_0_ × 100 (%), where V_0_ is the tumor volume at treatment start and V_t_ is the tumor volume at subsequent measurements.

At the end points, mice were euthanized with CO_2_ in compliance with protocols approved by the Institutional Animal Care and Use Committee (IACUC). Specific conditions for each xenograft model were described as follows:

CDX studies:

NCI-H441 xenograft model: The study was conducted by Shanghai MEDICILON Inc. and initiated by subcutaneously injected of 4 × 10^6^ cells into female Balb/c Nude mice, with 7 mice/group. Treatments started when the mean tumors volume reached an average of 150 mm^3^. Mice were randomized by tumor volume and body weight on Day 0, then received 2 doses at Day 0 and Day 7 of vehicle (0.9% NaCl) or SDP0505 at 3 and 10 mg/kg and U3-1402 analog at 3 mg/kg via intravenous injection.

PDX studies:

LUAD-OR1, LUAD-OR2 and CRC PDX models were conducted by LIDE Biotech. Tumor fragments (40–75 mg/mouse) were subcutaneously implanted into the right flank of NU/NU mice along with 20 μL Matrigel (Corning). Mice were monitored for tumor growth when the mean tumor volume reached an average of 150 mm^3^, mice were randomized into treatment groups, with this day defined as Day 0. For LUAD-OR1 and CRC PDX models, mice were then administered intravenously with vehicle, SDP0505 (at 3 and 10 mg/kg) and U3-1402 analog (at 3 mg/kg) on Day 0 and Day 7, respectively. For LUAD-OR2 PDX model, single dose of with vehicle, SDP0505 (at 3 and 10 mg/kg) and U3-1402 analog (at 3 mg/kg) was given on Day 0.

### Pharmacokinetic (PK) study in cynomolgus monkeys

The pharmacokinetic studies were performed by InnoStar Bio-tech Nantong Co. Ltd in compliance with protocols approved by the Institutional Animal Care and Use Committee (IACUC).

In this study, 18 cynomolgus monkeys were randomly assigned into three group (n = 6 per group, 3 males and 3 females). Each group received a single intravenous infusion of SDP0505 at doses of 1, 3, and 9 mg/kg over a period of 0.5 h. Blood samples were collected pre- and post-dose, with serum and plasma isolated at designated time points.

Serum concentrations of SDP0505 ADC and total antibody (TA) in the serum were quantified using a validated ELISA analysis. Specifically, for ADC detection, microplates were coated with a biotin-labeled anti-DXh antibody (named HRP02918, in-house developed). After blocking and washing, serum samples, along with calibration standards and quality controls, were added and incubated. Following another wash step, a Human IgG-heavy and light chain monkey-absorbed antibody (BETHYL, A80-319P) and TMB substrate were added for signal detection. ADCs with DAR ≥1 were measured. For TA detection, microplates were coated with human HGF receptor with his tag (Acro Biosystems, c-Met-H5227), and detection was performed using an HRP-conjugated anti-Human IgG (BETHYL, A80-319P). Pharmacokinetic parameters for each group were calculated using non-compartmental models. DXh concentrations in the Plasma samples were determined with a validated LC–MS/MS analysis after samples pre-treated with liquid–liquid extraction. Pharmacokinetic parameters were calculated using non-compartmental models.

### Repeat dose toxicity study in cynomolgus monkey

Forty cynomolgus monkeys (5 M/5F per group) received intravenous injection of SDP0505 at 0 (vehicle), 10, 20, or 40 mg/kg every two weeks for three doses (Day 1, 15, 29), followed by a 4-week recovery phase. Throughout the study, pivotal parameters were monitored and recorded, including clinical observations, hematology and serum chemistry. Gross necropsy followed with histopathology analysis were performed at endpoint of dosing phase and recovery phase.

### Statistical analysis

Statistical analyses were performed using GraphPad Prism or Excel, employing either one-way ANOVA or Student's t-test as appropriate. Significance levels are denoted as follows: ns (not significant, *P* > .05), ^*^P < .05, ^**^P < .01, and ^***^P < .001. All numerical data were presented as mean ± SEM unless otherwise indicated.

## Results

### HER3 and c-Met are co-expressed in EGFR TKI resistant tumors

**Figure 1:**
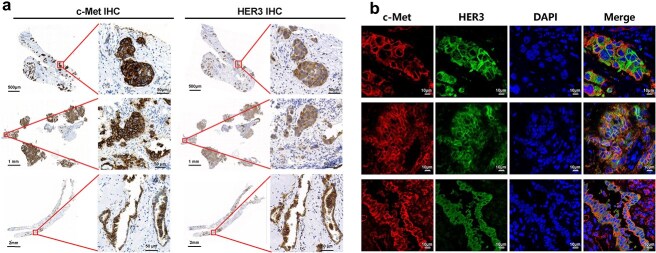
Co-expression of HER3 and c-Met in tumors. (a) Spatial co-localization of HER3 and c-Met in tumor samples from EGFR TKI-resistant NSCLC patients. Representative IHC staining of c-Met (left panels) and HER3 (right panels) expression in two consecutive tissue sections of three tumor samples. (b) Multiplex immunofluorescence (IF) was performed to confirm co-localization of HER3 and c-Met within the same tumor cells. Representative IF staining of c-Met, HER3, and DAPI expression in the same tissue sections of the three tumor samples were presented. Scale bar, 10 μm.

Emerging evidence indicates that acquired resistance to EGFR TKIs is frequently associated with the upregulation of HER3 or c-Met expression [5, 6]. However, whether HER3 and c-Met are co-expressed in EGFR TKI-resistant tumors remains unclear. To address this, we investigated their co-expression in tumor samples from five EGFR TKI-resistant NSCLC patients using HER3 and c-Met IHC. Remarkably, all five patients exhibited consistent co-expression patterns, as evidenced by spatially overlapping positive staining regions in consecutive tumor sections (representative figures shown in Fig. 1a). In addition, immunofluorescence-based co-localization analysis was further performed to confirm co-expression at the single-cell level, with representative images shown in Fig. 1b.

As the upregulation of HER3 and c-Met was reported not only in EGFR TKI-resistant adenocarcinoma of lung but also in other tumor types, we further evaluated the clinical relevance of HER3/c-Met co-expression in 265 tumor specimens from patients with different tumor types, including EGFR-mutant LUAD, EGFR-wildtype LUAD, LUSC, CRC, and GC. Analysis showed that HER3/c-Met co-positivity (H-score > 0) exceeded 67% across all five cancer types. Notably, HER3/c-Met exhibited particularly high co-expression (both H-scores ≥100) in 95% of CRC cases and 58% of EGFR-mutant LUAD cases (Supplementary Fig. S1). These findings demonstrated frequent HER3/c-Met co-expression across tumor types, supporting their potential as a dual target for BsADC.

### Optimal affinity selection of HER3 and c-Met arms in BsADC design

U3-1402, the first developed HER3-targeted ADC, has shown promising antitumor activity in clinical trials. However, only 29.8% of patients show a positive response, which is likely due to low HER3 abundance on tumor cells (10^3^–10^4^ receptors/cell) [14, 15]. To overcome this limitation, we propose a BsADC dual-targeting HER3 and c-Met. By incorporating c-Met as an additional target, we expect to increase the total number of available receptors on tumor cell surfaces, thereby enhancing ADC internalization and antitumor efficacy.

**Figure 2:**
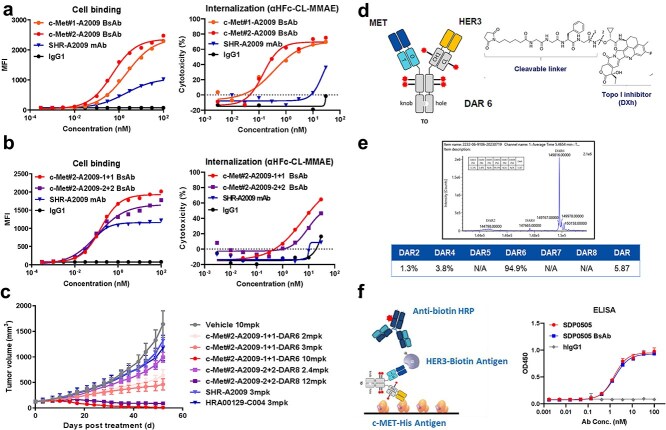
The screening and development of SDP0505. (a) Left: FACS-based binding assays of c-Met#1-A2009 BsAb, c-Met#2-A2009 BsAb and SHR-A2009 mAb in HER3/c-Met double-positive PC-9 cells. Right: Quantitative assessment of antibody internalization efficiency by conjugating to αHFc-CL-MMAE. HER3/c-Met double-positive PC-9 cells were treated with escalating concentrations of c-Met#1-A2009 BsAb conjugating to αHFc-CL-MMAE, c-Met#2-A2009 BsAb conjugating to αHFc-CL-MMAE, and SHR-A2009 mAb conjugating to αHFc-CL-MMAE to quantitative evaluation of internalization activity. (b) Left: FACS-based binding assays of c-Met#2-A2009-1 + 1 BsAb, c-Met#2-A2009-2 + 2 BsAb and SHR-A2009 mAb in HER3/c-Met double-positive PC-9 cells. Right: Quantitative assessment of different format antibody internalization efficiency by conjugating to αHFc-CL-MMAE. HER3/c-Met double-positive PC-9 cells were treated with escalating concentrations of c-Met#2-A2009-1 + 1 BsAb conjugating to αHFc-CL-MMAE, c-Met#2-A2009-2 + 2 BsAb conjugating to αHFc-CL-MMAE and SHR-A2009 mAb conjugating to αHFc-CL-MMAE to quantitative evaluation of internalization activity. (c) *In vivo* anti-tumor efficacy of c-Met#2-A2009-1 + 1 ADC (DAR6) and c-Met#2-A2009-2 + 2 ADC (DAR8) in a PC-9 xenograft model. Mice were intravenous injection with c-Met#2-A2009-1 + 1 ADC (DAR6) at 2, 3, or 10 mg/kg, c-Met#2-A2009-2 + 2 ADC (DAR8) at 2.4, 12 mg/kg and SHR-A2009, HRA00129-C004 at 3 mg/kg, once weekly for 8 doses. Tumors were measured twice a week, and the mean tumor volume ± SEM (mm^3^) was plotted against time. (d) Schematic structure of c-Met#2-A2009-1 + 1-DAR6 (SDP0505). SHR9265 is an exatecan derivative with a chiral cyclopropyl at the carbonyl alpha position. It was conjugated with c-Met#2-A2009-1 + 1 via a maleimide glycine–glycine-phenylalanine-glycine (GGFG) peptide linker to constitute a HER3 × c-Met BsADC coded SDP0505. The linker-payload was coupled to cysteine residues using a conventional strategy. Site 1: Intracellular cleavage site. Site 2: Hydrolysis reaction position. (e) DAR and its distribution profile of SDP0505 were determined via native mass spectrometry. (f) The dual-antigen sandwich ELISA assay was used to evaluate the simultaneous binding capability of SDP0505 and SDP0505 BsAb (a naked antibody) to human HER3 and c-Met proteins.

We previously developed SHR-A2009, a HER3-targeted ADC that showed remarkable antitumor efficacy in EGFR TKI-resistant NSCLC patients at a dose of 9.0 mg/kg Q3W, achieving an objective response rate (ORR) of 46.9% [16]. Building on this HER3-targeting scaffold, the medium-to-high affinity c-Met sequence #1 and the high-affinity c-Met sequence #2 were chosen as two bispecific antibody candidates for developing an optimal BsADC (Supplementary Fig. S2a). In HER3/c-Met double-positive PC-9 cells, we systematically compared cellular binding and internalization-mediated cytotoxicity between these two bispecific antibody candidates. To assess internalization, we employed αHFc-CL-MMAE, a polyclonal antibody conjugate that binds the Fc region of human IgG and releases MMAE upon lysosomal cathepsin cleavage following antibody-receptor internalization. This approach enabled quantitative evaluation of internalization activity via cytotoxicity readouts. The c-Met#2-A2009 BsAb construct exhibited 4-fold stronger cellular binding affinity and 3-fold greater cytotoxicity than c-Met#1-A2009 BsAb (EC_50_ = 0.52 nM vs. 2.16 nM, 0.14 vs. 0.36 nM, respectively), designating sequence #2 as the lead candidate (Fig. 2a). Both bispecific antibodies achieved 2-fold higher maximal binding capacity (E_max_) compared to the monospecific SHR-A2009 mAb, confirming that dual-targeting enhances cell surface antigen engagement. Moreover, when conjugated to αHFc-CL-MMAE, the bispecific antibodies showed superior *in vitro* antitumor efficacy over SHR-A2009 mAb (IC_50_ = 0.25 nM vs. 22.9 nM), underscoring the therapeutic advantage of this dual-targeting strategy.

### Strategic selection of antibody structural configuration

Although numerous bispecific antibody formats have been developed, BsADCs primarily utilize either 1 + 1 or 2 + 2 structural configurations [17–21]. Our comprehensive analysis demonstrated that BsADCs consistently exhibited 2-fold higher E_max_ values in double-positive cells compared to monospecific controls, regardless of format (Fig. 2b), validating the antigen density enhancement design. Interestingly, the 2 + 2 format displayed moderately reduced cellular binding and internalization efficiency relative to the 1 + 1 format, likely attributable to steric hindrance compromising target accessibility.

Given the distinct molecular mass profiles and maximum achievable DAR across formats, we proceeded to evaluate the antitumor activity of various structural formats at their respective maximal payload capacities, despite that 1 + 1 format had demonstrated superior binding and internalization. We conjugated DXh, our proprietary topoisomerase I inhibitor payload currently used in multiple clinical-stage ADCs [16, 22, 23] to both formats at their respective maximum DAR capacities. *In vitro* cytotoxicity assays revealed that the 2 + 2 format's higher payload capacity (DAR8) compensated for its weaker binding, resulting in improved potency (Supplementary Fig. S2b). However, *in vivo* studies showed that the 1 + 1 format achieved significantly better tumor growth inhibition (66% vs. 43% TGI) at equivalent payload doses (2 mg/kg for c-Met#2-A2009-1 + 1-DAR6 vs. 2.4 mg/kg for c-Met#2-A2009-2 + 2-DAR8), despite its lower drug loading (Fig. 2c). In this model, monotherapy with either HRA00129-C004 (c-Met-DXh, DAR 6, currently in Phase 2) [24] or SHR-A2009 showed limited antitumor activity (TGI = 31% and 21%, respectively). In contrast, the 1 + 1-DAR6 format bispecific ADC exhibited substantially enhanced efficacy (TGI = 66% and 78% at 2 and 3 mg/kg, respectively). These results indicated that the dual-targeting strategy outperforms single-target ADCs in this model,

### SDP0505 was characterized as a BsADC dual-targeting HER3 and c-Met with homogeneous DAR

Based on the results above, c-Met#2-A2009-1 + 1-DAR6 was selected as the lead candidate (designated SDP0505; structure shown in Fig. 2d). To eliminate light chain mispairing, we incorporated a Titin T chain/Obscurin-O chain (TO) into the Fab constant region [25], which disrupts an interchain disulfide bond and constrains maximum DAR to 6. This strategic modification yielded highly homogeneous DAR6 conjugates (Fig. 2e) with excellent plasma stability (<2% payload release after 21-day incubation at 37°C, Supplementary Fig. S2c). In addition, ELISA demonstrated fully preserved dual-antigen binding (HER3 and c-Met), with EC_50_ values equivalent to unconjugated antibodies (Fig. 2f), confirming that the conjugation process maintains native target engagement properties.

### In vitro antiproliferation activity of SDP0505

**Figure 3:**
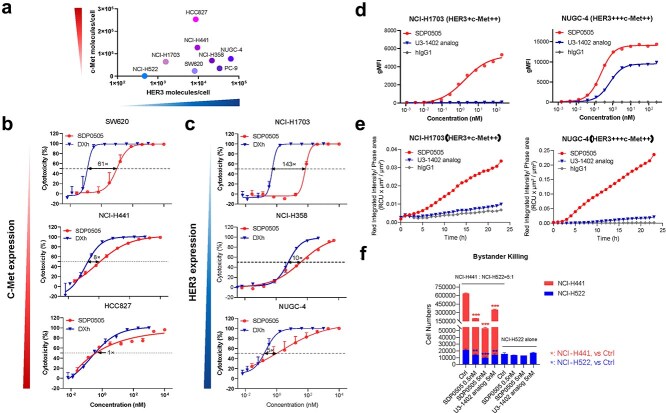
The antiproliferation activity and mechanism of action of SDP0505. (a) Quantitative expression profiles of HER3 and c-Met across seven tumor cell lines using FACS, presented using molecules/cell. (b) *In vitro* anti-proliferative activity of SDP0505 across tumor cell lines. Cell lines with comparable expression of HER3 and increasing expression of c-Met were treated with different concentration of SDP0505 and DXh for 9–11 days under 3D culture conditions. Cytotoxicity was evaluated using the CellTiter-Glo luminescent viability assay. (c) *In vitro* anti-proliferative activity of SDP0505 across tumor cell lines. Cell lines with comparable expression of c-Met and increasing expression of HER3 were treated with different concentration of SDP0505 and DXh for 9–11 days under 3D culture conditions. Cytotoxicity was evaluated using the CellTiter-Glo luminescent viability assay. (d) FACS-based binding assays of SDP0505, U3-1402 analog and SDP0505 in NCI-H1703 and NUGC-4 cell lines with different target expression profiles. (e) Internalization activity of SDP0505, U3-1402 analog and SDP0505 were evaluated using the Incucyte® system in NCI-H1703 cells and NUGC-4 cell lines with Incucyte® Human Fabfluor-pH Red Antibody Labeling Dye. (f) Bystander killing effect of SDP0505 and U3-1402 analog. SDP0505-sensitive cells NCI-H441 (high expression of both HER3 and c-Met) and SDP0505-insensitive cells NCI-H522 (double-negative expression of HER3 and c-Met) were labeled with CTV and CFSE, respectively, and inoculated at a 5:1 ratio (with NCI-H522 cells alone in control wells). The cells were treated with different concentrations of SDP0505 and U3-1402 analog for 5 days. After harvesting, cell counts were performed, and flow cytometry was used to analyze the proportions of CTV- and CFSE-labeled cells to determine the numbers of NCI-H441 and NCI-H522 cells. Statistical analysis was performed by one-way ANOVA, where ^**^P < .01 and ^***^P < .001.

For systematic characterization of SDP0505, we first established quantitative expression profiles of HER3 and c-Met across seven tumor cell lines using BD Quantibrite™ Beads-based flow cytometry. Quantitative analysis results revealed a low expression level of HER3 (10^3^–10^4^ molecules/cell), while a significantly higher expression of c-Met (10^4^–10^5^ molecules/cell; ~10-fold difference), aligning with published literatures (Fig. 3a) [14, 15]. The *in vitro* cytotoxic activity of SDP0505 was further evaluated across seven dual antigen-expressing cell lines under 3D culture conditions. SDP0505 demonstrated potent antitumor efficacy, with IC_50_ values ranging from 0.39 to 70.26 nM, showing superior or comparable activity when compared to the U3-1402 analog (Supplementary Table S1). Among the tested cell lines, SW620, NCI-H441 and HCC827 exhibited similar HER3 expression but progressively higher c-Met levels. In these three cell lines, lower SDP0505 IC_50_ values were observed in cells with higher c-Met expression, accompanied with a decreased IC_50_ shift ratio compared to the payload DXh (Fig. 3b). Conversely, in NCI-H1703, NCI-H358, and NUGC-4 cells, which showed comparable c-Met expression but progressively increasing HER3 expression levels, a progressive decrease in IC_50_ shift ratio compared to DXh was also observed. These results indicate that elevated expression of either c-Met or HER3 enhances the cytotoxic activity of dual-targeting ADC in double-positive tumor cells.

### Mechanistic investigation of SDP0505's anti-tumor activity

To characterize the mechanistic basis of SDP0505 activity, we systematically analyzed its binding properties across cell lines with different expression profiles (Fig. 3c). In NCI-H1703 cells (HER3 low, density = 1559 molecules/cell), the monospecific ADC U3-1402 analog showed negligible binding, whereas the bispecific SDP0505 exhibited potent binding (EC_50_ = 1.842 nM). This differential activity was further evident in NUGC-4 cells with moderate-to-high expression levels of both HER3 and c-Met, where SDP0505 demonstrated significantly enhanced cellular binding compared to the U3-1402 analog (EC_50_ = 0.23 nM vs. 0.69 nM). Notably, although SDP0505 exhibited reduced monovalent affinity for HER3 and only engaged one HER3 binding arm during cellular interaction compared to the U3-1402 analog, the incorporation of c-Met targeting dramatically improved the overall cell binding activity in dual antigen-expressing cells. After surface antigen engagement, the ADC undergoes receptor-mediated internalization and lysosomal processing, where the cleavable GGFG linker is cleaved to release the cytotoxic payload. Comparative analysis revealed that the U3-1402 analog showed limited internalization in both NCI-H1703 and NUGC-4 cells, while SDP0505 demonstrated markedly enhanced cellular uptake through its additional c-Met binding capability (Fig. 3d).

Bystander effect evaluation demonstrated that SDP0505 treatment induced dose-dependent cytotoxicity in antigen-negative NCI-H522 cells when co-cultured with HER3/c-Met-high NCI-H441 cells (Fig. 3e). At comparable concentrations, SDP0505 exhibited a modest but non-significant enhancement in bystander killing relative to the U3-1402 analog. Furthermore, functional characterization demonstrated that SDP0505 did not induce significant ADCC or CDC activity (Supplementary Fig. S3), despite maintaining typical human IgG1 FcγR and C1q binding properties as confirmed by SPR binding assays (Supplementary Tables S2 and S3).

### In vivo antitumor activities of SDP0505 in xenograft models

**Figure 4:**
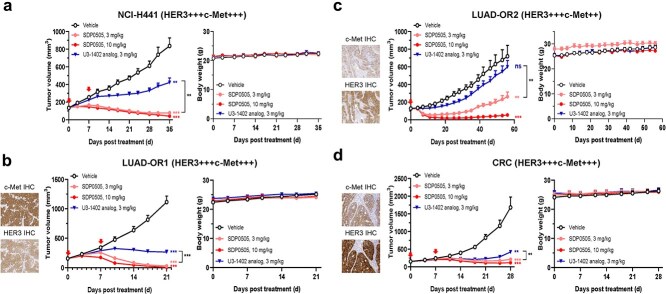
The *in vivo* anti-tumor activity of SDP0505 in CDX and PDX models. (a). (b). (c). (d). *In vivo* anti-tumor efficacy of SDP0505 and U3-1402 analog in an NCI-H441 CDX (a), LUAD-OR1 PDX (b), LUAD-OR2 PDX (c), and CRC PDX (d) model. Mice were intravenous injection at indicated time points with 3, or 10 mg/kg SDP0505 and 3 mg/kg U3-1402 analog. Tumors were measured twice a week, and the mean tumor volume ± SEM (mm^3^) was plotted against time (left), and the body weight of mice was recorded (right)*.* Expression level of c-Met and HER3 in PDX models were assayed by IHC. Statistical analysis was performed by student t-test based on tumor volume at the end of the study, where ns, *P* > .05, ^*^P < .05, ^**^P < .01, ^***^P < .001.

The *in vivo* anti-tumor efficacy of SDP0505 was initially evaluated using a subcutaneous xenograft model derived from the human NSCLC cell line NCI-H441 (Fig. 4a). When tumors reached ~150 mm^3^, mice were randomly grouped by tumor volume and intravenously injected with SDP0505 (weekly ×2 doses). At Day 35, SDP0505 demonstrated dose-dependent tumor growth inhibition, with 3 and 10 mg/kg doses showing highly significant efficacy versus vehicle control (*P* < .001; TGI: 91% and 95%, respectively). At the dose of 3 mg/kg, SDP0505 exhibited superior activity relative to U3–1402 analog at equivalent dose (TGI: 91% vs. 48%). No significant body weight loss was observed in any treatment groups throughout the study, indicating good tolerability.

PDX models with acquired resistance to standard therapies provide a clinically relevant platform for evaluating novel therapeutic strategies. *In vivo* comparisons of SDP0505 and U3-1402 analog in two established osimertinib-resistant PDX models (LUAD-OR1 and LUAD-OR2) were conducted to evaluate SDP0505's potential to overcome resistance. In the LUAD-OR1 model, SDP0505 demonstrated superior efficacy versus U3-1402 analog at 3 mg/kg (TGI: 97% vs 76%, *P* < .001), with complete responses in 1/6 and 5/6 animals at 3 and 10 mg/kg, respectively (Fig. 4b). Despite moderate HER3 expression (H-score = 190) in LUAD-OR2, U3-1402 analog exhibited minimal antitumor activity (TGI = 16%), whereas SDP0505 demonstrated significantly stronger efficacy (TGI = 64%). Furthermore, complete tumor regression occurred in one animal (1/6) at the 10 mg/kg dose group of SDP0505 (Fig. 4c).

Building upon target co-expression profiles and promising *in vitro* cytotoxicity data, we also investigated SDP0505’s therapeutic potential in CRC. In the CRC PDX model, SDP0505 demonstrated dose-dependent anti-tumor efficacy, achieving TGI rates of 87% (3 mg/kg) and 93% (10 mg/kg), respectively. The U3-1402 analog also resulted in a TGI rate of 75% (Fig. 4d). All treatment groups maintained normal body weight gain without drug-related toxicity, indicating favorable tolerability at the tested doses. Taken together, these results substantiated the potent activity of SDP0505 against EGFR TKI-resistant tumors and supported its clinical development for CRC.

### Pharmacokinetics and safety profile of SDP0505 in cynomolgus monkeys

**Figure 5:**
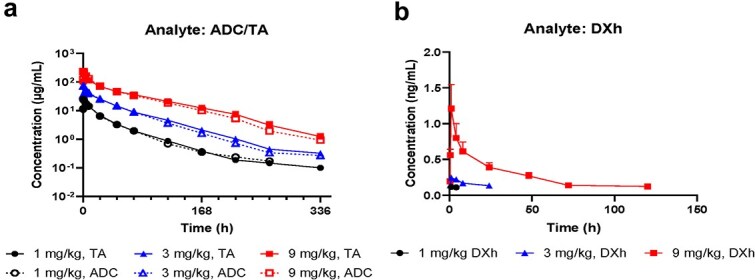
The pharmacokinetic properties of SDP0505 in cynomolgus monkeys. (a). (b). Cynomolgus monkeys were intravenously administered with 1, 3, 9 mg/kg SDP0505 on D0. Intact ADC and total antibody in monkey serum were measured by ELISA (a). Releasing payload in monkey plasma and tumor (SHR9265) was measured by LC–MS/MS (b).

SPR analysis demonstrated that SDP0505 could bind to HER3 proteins across multiple species but showed no detectable binding to rodent (rat/mouse) c-Met proteins (Supplementary Tables S4 and S5), establishing Cynomolgus monkeys as the pharmacologically relevant species for comprehensive pharmacokinetic and toxicological evaluation. Following single-dose intravenous administration of SDP0505 (1, 3, and 9 mg/kg) in naïve Cynomolgus monkeys, the exposure (C_max_ and AUC_last_) of both ADC and TA exhibited dose-proportional or slightly supra-proportional increases in serum (Fig. 5a). The ADC and TA exhibited comparable elimination profiles, with mean terminal half-lives of 43.2–50.0 h and 39.7–53.0 h, respectively. Importantly, the concentrations of free payload (DXh) in plasma were markedly lower than corresponding ADC levels in serum (C_max_ = 1.3 ng/mL vs. 236 μg/mL), confirming excellent *in vivo* stability of SDP0505 (Fig. 5b). Anti-drug antibodies (ADA) were detected in all dose groups which accelerated terminal clearance and reduced the half-life of ADC and TA, but had little impact on overall drug exposure.

In the GLP compliant repeat-dose toxicity study, Cynomolgus monkeys were intravenously administrated with SDP0505 at 10, 20, and 40 mg/kg every two weeks for five consecutive weeks (3 doses in total), followed by a 4-week recovery period. The highest non-severely toxic dose (HNSTD) was established at 40 mg/kg, with no mortality or moribund observed during the study (Supplementary Table S6). The treatment-related clinical pathology findings were characterized by decreased reticulocyte counts (RET) in both sexes and reduced albumin (ALB) levels in male animals. These statistically significant changes occurred only in the high-dose group at the end of dosing period, indicating a favorable toxicity profile. The thymus emerged as the principal toxic target organ of SDP0505, with minimal to mild thymic lymphocytopenia in both sexes at the high-dose group. Additionally, isolated cases of mild gastric mucosal regeneration (1 female) and jejunal mucosal regeneration (1 female) were noted in mid/high-dose groups respectively; the association between the gastrointestinal toxicity and SDP0505 was not definite due to the low incidence rate. All changes above were fully reversible by the recovery period endpoint.

## Discussion

Based on the colocalization results at the single-cell level, as well as IHC staining results from 270 clinical tumor specimens, we identified a high frequency of HER3 and c-Met co-expression in EGFR-mutant LUAD, CRC, and particularly in EGFR TKI-resistant NSCLC. These findings prompted us to develop a BsADC targeting both HER3 and c-Met that surpasses HER3-targeted ADCs and exhibits robust antitumor activity in EGFR TKI-resistant NSCLC and beyond. Leveraging our proprietary ADC platform, we engineered SDP0505 using an optimized exatecan-derived payload (DXh) conjugated to a balanced 1 + 1 bispecific antibody with equivalent target affinity. SDP0505 exhibited superior efficacy against TKI-resistant NSCLC compared to HER3-targeted monospecific ADC through enhanced receptor engagement and internalization. SDP0505 demonstrated favorable pharmacokinetic characteristics and acceptable safety profiles in cynomolgus monkeys.

HER3 has been considered as an undruggable target due to its lack of intrinsic kinase activity [26]. The emergence of U3-1402 has brought new insight for HER3-targeted ADC therapy, demonstrating rapid internalization properties *in vitro* that enable efficient killing in HER3-expressing tumor cells [27]. Although U3-1402 has shown encouraging efficacy in EGFR-mutant NSCLC, ~70% of patients remain unresponsive regardless of HER3 expression levels [7]. In May 2025, Daiichi Sankyo and Merck voluntarily withdrew the BLA for U3-1402 in previously treated locally advanced or metastatic EGFR-mutated NSCLC, following the lack of OS benefit in the HERTHENA-Lung02 trial. Given that HER3 internalization activity positively correlates with receptor density [28], we hypothesize that low abundance of HER3 receptors might limit the therapeutic efficacy by restricting intracellular drug delivery. To address these limitations, our therapeutic strategy focuses on developing a HER3-directed bispecific agent that simultaneously engages a complementary target meeting three critical criteria: (1) high tumor cell surface expression, (2) robust intrinsic internalization capacity, and (3) clinical validation of target relevance. The selection of c-Met is not only because it exhibits genomic amplification and protein upregulation post-EGFR TKI treatment, but also partially supported by emerging clinical validation of amivantamab’s proof-of-concept efficacy in EGFR TKI-resistant NSCLC. Although prior studies have documented co-expression of HER3 and c-Met in human colorectal cancer and shown that combined HER3 antibody and c-Met small-molecule inhibition effectively reduces tumor growth in colorectal cancer xenograft models [29], the co-expression profile of HER3 and c-Met in EGFR TKI-resistant patients remains unexplored. Here, we report for the first time the co-expression and co-localization of HER3 and c-Met in this resistant population (Fig. 1), providing a scientific rationale for dual targeting of these molecules as a therapeutic strategy. Moreover, Engelman et al. demonstrated that MET amplification confers resistance to the first-generation EGFR TKI gefitinib through activation of HER3–PI3K/AKT bypass signaling [30]. This finding offers a potential mechanistic explanation for the co-expression observed in TKI-resistant patients. By simultaneously targeting two complementary resistance pathways, this approach could surpass the efficacy constraints of HER3 monospecific ADC and amivantamab in TKI-resistant patients, enabling sustained tumor growth suppression in a broaden population. In addition, dual targeting of HER3 and c-Met may reduce the risk of EGFR-related skin disorders compared to amivantamab and BL-B01D1 in clinical settings. In addition, the co-expression pattern of HER3 and c-Met in CRC and GC (Fig. 1b) further supports the potential application of the BsADC for other solid tumors.

Blinatumomab, the first bispecific antibody therapy approved by FDA, has ushered in a new era of bispecific antibody development [31]. To date, over 100 bispecific antibody formats have been reported, including both Fc-based and fragment-based architectures, many of which have advanced into clinical stage [18]. In contrast to bispecific antibodies, the development of BsADCs remains in its infancy. Current clinical candidates predominantly employ 1 + 1 or 2 + 2 IgG1-based formats, as these configurations demonstrate optimal antibody stability, minimize ADC aggregation post-conjugation, and provide suitable conjugation sites [10, 17–21]. Leveraging the HER3 sequence from our phase III candidate SHR-A2009, we developed BsADCs through systematic screening of cell binding and internalization assays. All tested configurations (incorporating either c-Met#1 or c-Met#2 in 1 + 1 and 2 + 2 formats) showed higher E_max_ values than SHR-A2009 mAb (Fig. 2a and b), confirming that the dual-targeting strategy enhanced cell surface antigen engagement. When conjugated with αHFc-CL-MMAE, BsADCs demonstrated superior antitumor activity versus monospecific HER3 ADC, supporting enhanced internalization activity through simultaneous HER3/c-Met targeting (Fig. 2a and b). The balanced affinity between HER3 and c-Met#2 arms, combined with the high intrinsic internalization capacity of both arms, enables enhanced cell binding and internalization activity in dual-antigen positive cells. Notably, the 1 + 1 bispecific format exhibited higher cell binding and internalization activity than the 2 + 2 format, likely due to its structural simplicity, which reduced steric hindrance and facilitated more efficient lysosomal trafficking. This design prioritizes functional payload delivery over maximal drug loading, as evidenced by the superior *in vivo* efficacy of the 1 + 1 format (66% TGI) over the 2 + 2 variant (43% TGI) at equivalent toxin doses (Fig. 2c). Furthermore, with lower molecular weight (60% of the 2 + 2 format), the 1 + 1 format may improve tumor penetration, thereby enhancing therapeutic efficacy.

DAR optimization remains pivotal in ADC design, balancing payload potency with toxicity profiles. While early ADCs employing microtubule inhibitors favored lower DARs (2–4) to mitigate toxicity, the paradigm has shifted toward topoisomerase I inhibitors (e.g. DS-8201) enabling higher DARs (6–8) for improved efficacy without significant target-mediated toxicity, as exemplified by HER3-targeting ADC U3-1402 at DAR8 [7]. In contrast, c-Met expression in normal epithelial cells and hepatocytes necessitates a lower DAR 6 for c-Met-targeting ADCs [14, 32]. Despite this precaution, c-Met-related toxicities (e.g. peripheral edema and hypoalbuminemia) persist in clinical trials [33]. To address this challenge, SDP0505 employs a DAR6 design with a monovalent binding format to reduce off-target c-Met binding in normal tissues and improve clinical tolerability. Simultaneously, conjugation technology evolution addresses DAR heterogeneity. Traditional maleimide-cysteine methods produce stochastic payload distributions across IgG1's eight solvent-accessible cysteines, whereas engineered platforms like Enhertu's exhaustive cysteine conjugation achieve uniform DAR8. Building on these principles, our BsADC innovatively replaces CH1-CL domains with Obscurin-O/Titin-T chain interactions, not only prevents light-chain mispairing but also reduces reducible cysteines from eight to six, yielding >95% homogeneous DAR6 conjugates (Fig. 2e) with exceptional plasma stability (<2% payload release over 21-days incubation at 37°C; Supplementary Fig. S2c). This synergistic design reconciles the conflicting demands of target-specific DAR optimization and conjugation homogeneity critical for next-generation ADCs.

The enhanced cell binding, internalization and antiproliferation activity of SDP0505 against U3-1402 analog in HER3/c-Met co-expressing cell lines also elaborates the therapeutic advantage of dual antigen engagement. The difference in cytotoxicity between SDP0505 and the payload DXh decreased with higher c-Met or HER3 expression levels, suggesting that both c-Met and HER3 engagement contributes to the enhanced killing of dual-positive tumor cells. This aligns with quantitative flow cytometry data showing ~10-fold higher c-Met expression than HER3, which compensates for HER3’s limitations by increasing total antigen density (Fig. 3). The robust *in vivo* efficacy of SDP0505 across multiple preclinical models, including third-generation EGFR TKI-resistant PDXs, reveals its dual-targeting mechanism and underscores the potential to overcome the standard of care (SOC) refractory cancer types. Consistent with the *in vitro* assays, SDP0505 also outperformed U3-1402 analog *in vivo* when dosed at the same dose (3 mg/kg), reinforcing the therapeutic advantage of BsADC over conventional monospecific ADCs. Strikingly, in the U3-1402-resistant LUAD-OR2 model (TGI = 16% with U3-1402 analog despite moderate HER3 expression), SDP0505 achieved 64% tumor growth inhibition at the same dose (*P* < .01 vs U3-1402 analog). These results suggest SDP0505 may provide clinical benefit in U3-1402-refractory populations, highlighting its potential to address unmet therapeutic needs (Fig. 4). In addition to the superior antitumor activity compared to a HER3-targeting ADC, SDP0505 also exhibited enhanced efficacy compared to HRA00129-C004 (c-Met-DXh, DAR 6, currently in Phase 2) [24] in the PC-9 CDX model (Fig. 2c). Telisotuzumab vedotin, a c-Met targeting ADC, received FDA accelerated approval in 2025 based on positive results from the LUMINOSITY study, which reported an ORR of 35% in patients with locally advanced or metastatic non-squamous EGFR wild-type NSCLC exhibiting high c-Met expression [34]. However, its clinical benefit is largely confined to tumors with high c-Met expression, a subset that accounts for only ~13.5% of non-squamous EGFR wild-type NSCLC patients [35]. In contrast, SDP0505 is being developed not only for EGFR-mutant NSCLC, but also for EGFR wild-type NSCLC, GC, and CRC. For EGFR wild-type patients with moderate or low c-Met expression, who are less likely to benefit from c-Met-targeted monotherapy, the inclusion of a HER3 arm in SDP0505 may offer improved antitumor efficacy and thereby extend therapeutic benefit to a wider patient population. In addition, although compelling *in vivo* efficacy has been observed in EGFR-mutant NSCLC, clinical translation of SDP0505 faces additional layers of complexity, including tumor heterogeneity, microenvironmental factors, and the dynamics of bystander effects. Future studies incorporating orthotopic models could provide a more physiologically relevant assessment of SDP0505 distribution and its potential effects on neighboring normal cells than subcutaneous models. Furthermore, the lack of a functional immune system in these models precludes evaluation of ADC-mediated bystander toxicity on immune cells. Therefore, the safety profile of SDP0505, particularly regarding immune-related effects, will be further evaluated in immunocompetent tumor-bearing mice.

The favorable pharmacokinetic and safety profiles of SDP0505 in cynomolgus monkeys further reinforced its clinical translatability. The dose-proportional exposure of both ADC and total antibody, coupled with minimal free payload release (<0.6% of total ADC concentration, Supplementary Fig. S2c), highlights exceptional *in vivo* stability, a critical attribute for reducing systemic toxicity while maintaining therapeutic payload delivery. The comparable elimination half-lives of ADC and total antibody (~43–50 h) suggest stable linker-payload retention (Fig. 5), consistent with the <2% premature payload release in the *in vitro* plasma stability assays. The toxicity profile of SDP0505 in cynomolgus monkeys, characterized by thymic lymphocytopenia and mild hematologic changes at the highest dose (40 mg/kg), aligns with the on-target mechanism of topoisomerase I inhibitor payloads that preferentially affect rapidly dividing cells. The reversible nature of all observed adverse effects further supports its clinical viability. Notably, no severe organ toxicity or mortality was observed even at doses substantially exceeding therapeutic levels (40 mg/kg in monkeys vs. efficacious doses of 3–10 mg/kg in mouse PDX models), indicating a wide therapeutic index. In repeat-dose toxicity studies, the HNSTD of SHR-A2009 (DAR4) in cynomolgus monkeys was established as 60 mg/kg – equivalent to the toxin load of SDP0505 at 40 mg/kg. This comparable payload exposure suggests that the incorporation of the c-Met-targeting arm does not increase toxicity relative to the monospecific ADC. These preclinical safety data, combined with the potent efficacy demonstrated in EGFR TKI-resistant models, position SDP0505 as a promising candidate for clinical development.

Collectively, we generated SDP0505, a potential first-in-class BsADC featuring balanced 1 + 1 antibody affinity for both c-Met and HER3, demonstrating efficient tumor cell internalization and a robust bystander effect. It exerted potent anti-tumor activities and acceptable safety profiles. An open-label, multicenter, phase I study of SDP0505 has been initiated in 2025 Q1 to investigate its safety, tolerability, pharmacokinetics, and efficacy in patients with advanced or metastatic solid tumors.

## Supplementary Material

ABT-2025-048-Supplementary_information-clean_tbag015

## Data Availability

Data supporting the findings of this study are available from the corresponding author Cheng Liao on request.

## Animal research statement

All animal experiments were conducted in accordance with a protocol approved by the Institutional Animal Care and Use Committee (IACUC) at the testing facility, and adhered to all applicable institutional guidelines for animal welfare.
