# Supplementary material for: Development of SDP0505: a first-in-class HER3 × c-Met bispecific ADC, demonstrates potent antitumor activity in EGFR TKI-resistant NSCLC, CRC, and beyond
Source: Antib Ther. 2026 Apr 21;9(2):201–13. doi: 10.1093/abt/tbag015 (PMC13175982; doi:10.1093/abt/tbag015)
Supplement: ABT-2025-048-Supplementary_information-clean_tbag015 [file abt-2025-048-supplementary_information-clean_tbag015.docx]

**Development of SDP0505: A First-in-Class HER3×c-Met Bispecific ADC, Demonstrates Potent Antitumor Activity in EGFR TKI-resistant NSCLC, CRC and Beyond**

Supplementary Figures


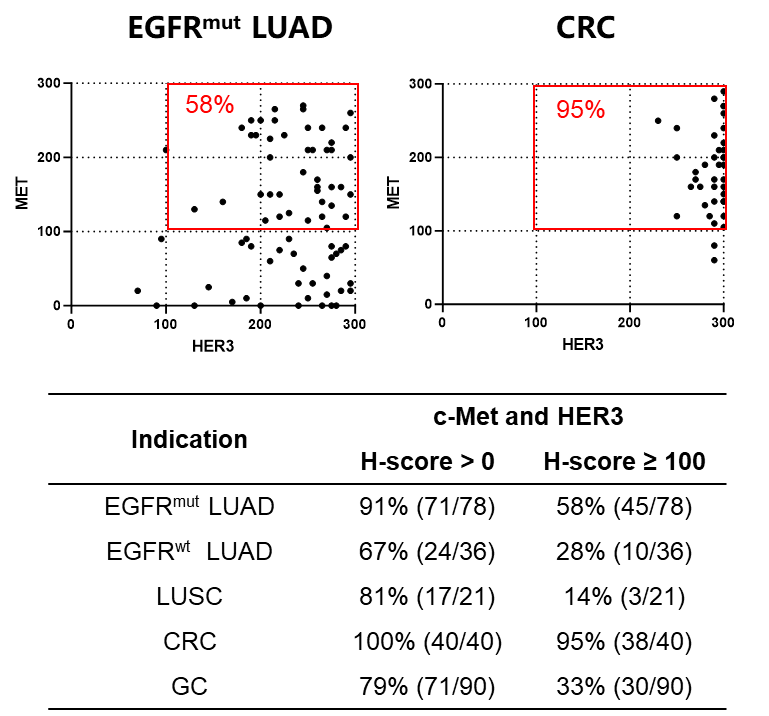


**Supplementary Fig. S1**. **Co-expression patterns of HER3 and c-Met across EGFR-mutant LUAD and other solid tumors.**

**Top:** scatter plot correlating HER3 and c-Met H-scores (range 0–300) in EGFR-mutant LUAD (n=78) and CRC (n=40). The red frame indicates co-expression thresholds (both H-score ≥100). **Bottom:** co-positivity (H-score>0) and co-expression (both H-scores ≥100) rates of HER3 and c-Met across tumor types.

**
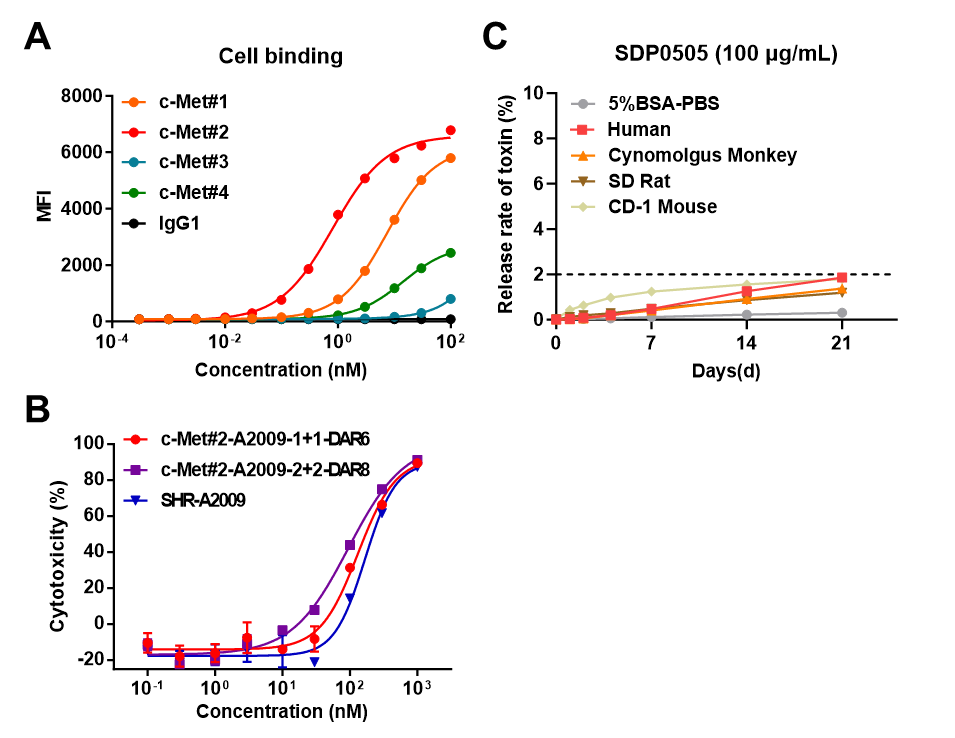
**

**Supplementary Fig. S2**. **The screening and identification of SDP0505.**

**(A)**. FACS-based binding assay of c-Met targeting antibodies (c-Met#1-#4) and human IgG1 control in NCI-H1975 cells.

**(B)**. Two-dimensional cytotoxicity assessment of c-Met#2-A2009-1+1-DAR6, c-Met#2-A2009-2+2-DAR8 and SHR-A2009 in PC-9 cells. Antibodies were conjugated to DXh at different DAR. ADCs were incubated with cells for 6 days followed by cell viability measurement using the CTG assay.

**(C)**. *In vitro* plasma stability of SDP0505 across species. SDP0505 (100 μg/mL) was incubated with plasma at 37℃ for 21 days, and free DXh was quantified by LC-MS/MS.


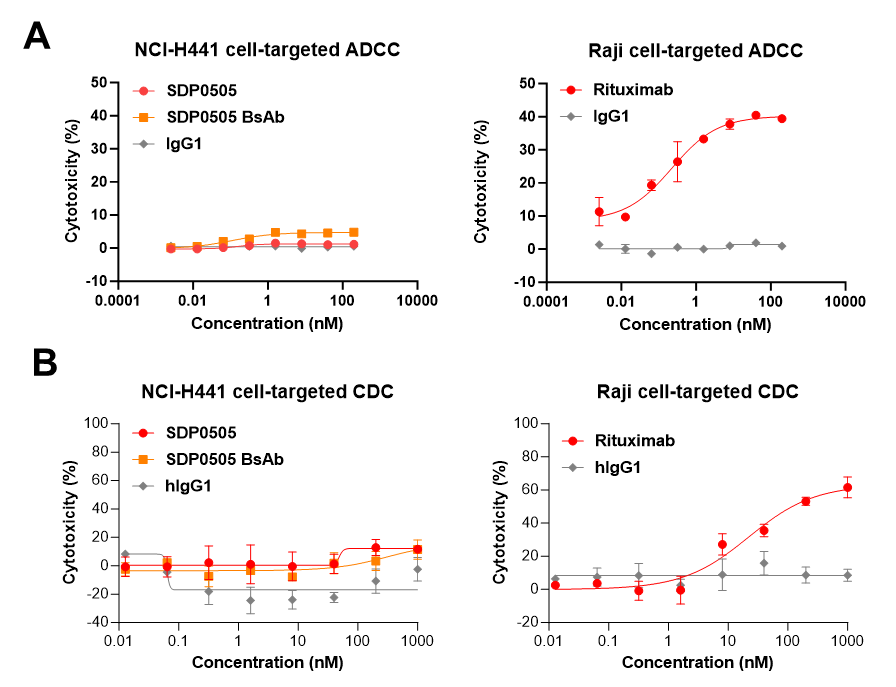


**Supplementary Fig. S3. ADCC and CDC effects of SDP0505.**

**(A)**. **Left:** ADCC activity of SDP0505, SDP0505 BsAb (naked antibody) and human IgG1. NCI-H441 was used as the target cells and NK92-CD16A-176V was used as the effector cells, with an effector-to-target ratio of 5:1. **Right:** ADCC activity of Rituximab and human IgG1. Raji was used as the target cells and NK92-CD16A-176V was used as the effector cells, with an effector-to-target ratio of 5:1.

**(B)**. **Left:** CDC activity of SDP0505, SDP0505 BsAb (naked antibody) and human IgG1 in NCI-H441 cells. **Right:** CDC activity of Rituximab and human IgG1 in Raji cells. Cells were treated with indicated drugs for 4 hours followed by cell viability assessment.

Supplementary Tables

**Supplementary Table S1. Antiproliferation activity of SDP0505 in tumor cells with different HER3 and c-Met expression.**

| **Cell lines** | **Tumor types** | **IC_50_ (nM)** | | |
| --- | --- | --- | --- | --- |
|  |  | **SDP0505** | **U3-1402 analog** | **DXh** |
| HCC827 | NSCLC | 0.39 ± 0.20 | 16.41 | 0.36 ± 0.11 |
| NCI-H441 | NSCLC | 1.39 ± 0.66 | 31.12 | 0.18 ± 0.05 |
| SW620 | CRC | 8.50 ± 7.22 | 120.4 | 0.14 ± 0.05 |
| NUGC-4 | GC | 0.59 ± 0.87 | 5.29 | 0.12 ± 0.09 |
| PC-9 | NSCLC | 51.30 ±26.49 | 83.59 | 0.23 ± 0.14 |
| NCI-H1703 | NSCLC | 69.52 ± 6.90 | 88.49 | 0.49 ± 0.15 |
| NCI-H358 | NSCLC | 70.26 ± 43.41 | 234.2 | 6.21 ± 2.36 |

The data for SPD01873 and DXh were derived from three independent experimental replicates, while results for the U3-1402 analog were obtained from a single biological repeat.

NSCLC, non-small cell lung cancer; CRC, colorectal cancer; GC, gastric cancer.

**Supplementary Table S2. The binding affinity of SDP0505 to human Fc receptors.**

| **Antigen** | **Test article** | **ka (1/Ms)** | **kd (1/s)** | **KD (M)** |
| --- | --- | --- | --- | --- |
| FcRn* | SDP0505 | / | / | 1.43E-07 |
| FcγRIIIa/CD16a (F176)* |  | / | / | 1.16E-05 |
| FcγRIIIa/CD16a (V176) |  | 7.93E+04 | 1.80E-02 | 2.26E-07 |
| FcγRIIIb/CD16b* |  | / | / | 1.55E-05 |
| FcγRIIa/CD32a (H167)* |  | / | / | 5.44E-06 |
| FcγRIIa/CD32a (R167)* |  | / | / | 6.46E-06 |
| FcγRIIb/hCD32b* |  | / | / | 1.18E-05 |
| FcγRI/hCD64 |  | 8.62E+04 | 8.47E-04 | 9.83E-09 |

*: Fitted using the Steady State Affinity model, only the KD value was obtained, with no Ka or Kd.

**Supplementary Table S3. The binding affinity of SDP0505 to human C1q proteins.**

| **Test article** | **ka (1/Ms)** | **kd (1/s)** | **KD (M)** |
| --- | --- | --- | --- |
| SDP0505* | / | / | 1.14E-07 |
| Human IgG1* | / | / | 4.18E-08 |

*: Fitted using the Steady State Affinity model, only the KD value was obtained, with no Ka or Kd.

**Supplementary Table S4. Binding of SDP0505 to HER3 protein from different species as determined by SPR assay.**

| **Antigen** | **Test article** | **ka (1/Ms)** | **kd (1/s)** | | | **KD (M)** |
| --- | --- | --- | --- | --- | --- | --- |
| Human HER3 | SDP0505 | 9.37E+05 | 1.17E-02 | | | 1.25E-08 |
|  | SDP0505 mAb | 1.07E+06 | 1.22E-02 | | | 1.14E-08 |
|  | U3-1402 analog | 2.30E+05 | | 4.23E-04 | 1.84E-09 | |
| Cynomolgus HER3 | SDP0505 | 8.58E+05 | 1.30E-02 | | | 1.51E-08 |
|  | SDP0505 mAb | 8.91E+05 | 1.32E-02 | | | 1.48E-08 |
|  | U3-1402 analog | 1.53E+05 | 4.64E-04 | | | 3.04E-09 |
| Rat HER3 | SDP0505 | 6.14E+05 | 1.11E-02 | | | 1.81E-08 |
|  | SDP0505 mAb | 6.88E+05 | 1.17E-02 | | | 1.70E-08 |
|  | U3-1402 analog | 1.12E+05 | 8.60E-03 | | | 7.65E-08 |
| Mouse HER3 | SDP0505 | 6.11E+05 | 1.07E-02 | | | 1.76E-08 |
|  | SDP0505 mAb | 9.79E+05 | | 1.17E-02 | 1.19E-08 | |
|  | U3-1402 analog | 9.50E+04 | | 8.92E-03 | 9.39E-08 | |

**Supplementary Table S5. Binding of SDP0505 to c-Met protein from different species as determined by SPR assay.**

| **Antigen** | **Test article** | **ka (1/Ms)** | **kd (1/s)** | **KD (M)** |
| --- | --- | --- | --- | --- |
| Human c-Met | SDP0505 | 1.44E+05 | 1.25E-03 | 8.72E-09 |
|  | SDP0505 mAb | 2.43E+05 | 1.23E-03 | 5.05E-09 |
| Cynomolgus c-Met | SDP0505 | 1.37E+05 | 2.21E-03 | 1.61E-08 |
|  | SDP0505 mAb | 1.95E+05 | 2.21E-03 | 1.13E-08 |
| Rat c-Met | SDP0505 | No binding | | |
|  | SDP0505 mAb |  |  |  |
| Mouse c-Met | SDP0505 | No binding | | |
|  | SDP0505 mAb |  |  |  |

**Supplementary Table S6. Main toxicity findings of SDP0505 in repeat-dose toxicity study in Cynomolgus monkeys**

| **Species** | **Cynomolgus monkeys** |
| --- | --- |
| **Regimens** | 0, 10, 20, 40 mg/kg  Intravenous, every 2 weeks for 1 months (3 times in total) |
| **No. of animals** | 3/sex/group (main): all dose groups  2/sex/group (recovery): all dose groups |
| **Lethal dose** | > 40 mg/kg |
| **Hematology** | 40 mg/kg: decreased RET (both sexes) |
| **Clinical Chemistry** | 40 mg/kg: decreased ALB (only in female) |
| **Target organs and tissues** | 20 mg/kg: gastrointestinal tract (1/6, mild gastric mucosal regeneration, end of dosing period)  40 mg/kg: thymus (6/6, minimal to mild lymphocytopenia, end of dosing period); gastrointestinal tract (1/6, jejunal mucosal regeneration, end of dosing period) |
| **HNSTD** | 40 mg/kg |
